# Review of Journal of Cardiovascular Magnetic Resonance 2012

**DOI:** 10.1186/1532-429X-15-76

**Published:** 2013-09-04

**Authors:** Dudley J Pennell, A John Baksi, John Paul Carpenter, David N Firmin, Philip J Kilner, Raad H Mohiaddin, Sanjay K Prasad

**Affiliations:** 1 Cardiovascular Biomedical Research Unit, Royal Brompton Hospital, Sydney Street, London, SW3 6NP, UK; 2 Imperial College, London, UK

## Abstract

There were 90 articles published in the Journal of Cardiovascular Magnetic Resonance (JCMR) in 2012, which is an 8% increase in the number of articles since 2011. The quality of the submissions continues to increase. The editors are delighted to report that the 2011 JCMR Impact Factor (which is published in June 2012) has risen to 4.44, up from 3.72 for 2010 (as published in June 2011), a 20% increase. The 2011 impact factor means that the JCMR papers that were published in 2009 and 2010 were cited on average 4.44 times in 2011. The impact factor undergoes natural variation according to citation rates of papers in the 2 years following publication, and is significantly influenced by highly cited papers such as official reports. However, the progress of the journal's impact over the last 5 years has been impressive. Our acceptance rate is approximately 25%, and has been falling as the number of articles being submitted has been increasing. In accordance with Open-Access publishing, the JCMR articles go on-line as they are accepted with no collating of the articles into sections or special thematic issues. For this reason, the Editors have felt that it is useful once per calendar year to summarize the papers for the readership into broad areas of interest or theme, so that areas of interest can be reviewed in a single article in relation to each other and other recent JCMR articles. The papers are presented in broad themes and set in context with related literature and previously published JCMR papers to guide continuity of thought in the journal. We hope that you find the open-access system increases wider reading and citation of your papers, and that you will continue to send your quality manuscripts to JCMR for publication.

## Vessel wall & MR angiography

CMR is used widely for research into the vessel wall It is most frequently used for the carotid arteries where it characterizes plaque content [1,2], vulnerability, atheroma burden, natural history of progression [3], and response to treatment [4]. However, imaging of the coronaries is also making progress. There are fewer reports of coronary luminal imaging, but reports continue of coronary wall imaging [5,6], and identification of coronary anomalies [7]. This is an area of CMR where 3T has had significant impact.

### Quantitative evaluation of high intensity signal on MIP images of carotid atherosclerotic plaques from routine TOF-MRA reveals elevated volumes of intraplaque hemorrhage and lipid rich necrotic core

High intensity signals in carotid plaques seen on TOF-MRA MIP images acquired at 3.0 Tesla was shown to be associated with increased intraplaque haemorrhage and lipid-rich necrotic core volumes [8]. This is an interesting finding and a simple approach to identify these clinically relevant plaques but the study is limited by poor sensitivity and the lack of histological validation.

### Characteristics of carotid atherosclerotic plaques of chronic lipid apheresis patients as assessed by In vivo high-resolution CMR - a comparative analysis

Morphology and composition of carotid plaques were evaluated in patients undergoing chronic lipid apheresis (LA) who suffered recent stroke using spin-echo images obtained at 3T [9]. Despite a more severe risk profile for cardiovascular complications in LA-patients, chronic LA is associated with lower cholesterol, LDL and LDL/HDL as well as significantly lower lipid content in carotid plaques compared to plaques of patients without LA with similar degrees of stenosis. However, the number of patients studied is small and the study design allows only a snapshot of plaque burden and composition at one single time point and no definite conclusions can be drawn on the evolution of plaque composition over time.

### High-resolution intravascular magnetic resonance quantification of atherosclerotic plaque at 3T

Intravascular CMR using 3T and loopless CMR detector was used to obtain in vitro and in vivo high resolution images of atherosclerotic plaques and the findings were compared with histology [10]. The authors showed that atherosclerotic lesions in human arterial specimens in vitro can accurately measure plaque fibrous cap thickness at high-resolution over a range of about 100-1000μm and that the same approach is feasible in vivo.

### Optimization of single shot 3D breath-hold non-enhanced MR angiography of the renal arteries

Further to previous papers in JCMR on MRA techniques [11], and multifaceted assessment of the kidneys [12], Tan et al. describe a single-shot 3D magnetization-prepared steady-state free precession technique was implemented to assess the renal arteries and compared with a more traditional navigator-based approach [13]. The initial results suggest a potential supplementary clinical role for the breath-hold technique in the evaluation of suspected renal artery diseases.

### Detection of thrombus size and protein content by ex vivo magnetization transfer and diffusion weighted MRI

Magnetization Transfer Contrast (MTC) and Diffusion Weighted Imaging (DWI) were used for ex vivo thrombus imaging at 11.7T. MTC estimated more accurately the thrombus area compared to T1W and T2W images the rabbit aortic specimens [14]. The quantitative measure of % MTR correlated with the protein content of the thrombus and allowed the discrimination between protein-rich (organized) and unorganized (cell-rich) thrombus. Although application of these techniques for in vivo thrombus imaging is clinically relevant but, ex vivo imaging at 11.7 T is of limited clinical interest as results are not transferable to clinical lower field strengths. Optimization of this work for 3T has been attempted but is quite weak in this study.

## CMR in inflammatory vasculitis

This is a well written and well illustrated review of CMR in inflammatory vasculitis with an extensive bibliography [15]. As such, this review is a useful reference to clinicians from a variety of disciplines.

### Cardiovascular magnetic resonance in systemic hypertension

The authors provide a comprehensive review of the current and emerging clinical and research applications of CMR in systemic hypertension which is a highly prevalent potentially modifiable cardiovascular risk factor [16]. CMR provides accurate and reproducible measures of ventricular volumes, mass, function and haemodynamics as well as uniquely assesses myocardial diffuse and focal fibrosis. In addition, CMR is well suited for the differential diagnosis of LV hypertrophy and the exclusion of common secondary causes for hypertension.

## Pulmonary hypertension

The clinical importance of pulmonary hypertension is being increasingly recognised and new treatments are being evaluated. Pulmonary embolism remains a major problem during in-patient episodes, and evaluation has been improved using computed tomography, although CMR also shows flow abnormalities which can be useful [17].

### Pulmonary endarteretomy normalizes interventricular dyssynchrony and right ventricular systolic wall stress

Interventricular mechanical dyssynchrony is a mark of pulmonary hypertension. The authors showed that L-R dyssynchrony in peak strain recovers to normal values after pulmonary endarterectomy in patients with chronic thromboembolic pulmonary hypertension [18]. The RV end-systolic wall stress plays a key role in this recovery, reflecting a complex interplay of pulmonary artery pressure, RV radius and wall thickness.

### Late gadolinium enhancement cardiovascular magnetic resonance predicts clinical worsening in patients with pulmonary hypertension

Late gadolinium enhancement (LGE) occurs at the right ventricular insertion point in patients with pulmonary hypertension and has been shown to correlate with CMR derived RV indices but the prognostic role of LGE is not well established. This study showed that LGE is a marker of advanced disease and poor prognosis [19]. RVEF was also found to be independent non-invasive imaging predictor of adverse outcomes in this patient population.

### Diagnostic accuracy of cardiovascular magnetic resonance imaging of right ventricular morphology and function in the assessment of suspected pulmonary hypertension results from the ASPIRE registry

CMR was shown to be a useful alternative to echocardiography in the evaluation of suspected pulmonary hypertension [20]. The results of this study supports a role for the routine measurement of ventricular mass index, late gadolinium enhancement and the use of phase contrast imaging in addition to right heart functional indices in patients undergoing diagnostic CMR evaluation for suspected pulmonary hypertension.

### Cardiovascular magnetic resonance in pulmonary hypertension

This is a comprehensive review of the role of CMR in assessment of patients with pulmonary hypertension [21]. CMR is especially useful in patients with a diagnosis of (or suspected of having) PAH associated with congenital heart disease and allows discrimination of other features which might point to an alternative aetiology. It is a flexible research tool which has opened new avenues for understanding treatment effects, outcomes and pathogenesis. It appears inevitable that its place in the management of PH will evolve as evidence supporting its use accumulates.

### Diffusion tensor imaging

Diffusion tensor imaging (DTI) is an MR technique which uses diffusion weighting to identify components of tissue architecture. It has most commonly been applied to the brain, where larger diffusion values occur along nerve fibres in comparison to across fibres. This is interpreted to mean that diffusion occurs more freely along nerve fibres than between nerve fibres. Spectacular colour coded images showing the nerve fibre directions and long distance connections in brain white matter can be generated. The technique can also be used for the heart [22], however most work has been done in animals [23], or in ex-vivo human hearts, because of the high difficulty of deriving DTI data from the moving heart. Recently a more robust imaging sequence for in-vivo human cardiac DTI has been described which makes human studies more approachable [24]. Cardiac DTI provides information on mean intravoxel myocyte orientation and potentially myocardial disarray, and therefore first interest has been in investigation of hypertrophic cardiomyopathy (HCM).

### Reproducibility of in-vivo diffusion tensor cardiovascular magnetic resonance in hypertrophic cardiomyopathy

The authors report the reproducibility of using a stimulated-echo single-shot-EPI sequence with zonal excitation and parallel imaging in 10 patients with HCM scanned on 2 different days [25]. Fractional anisotropy (FA), mean diffusivity (MD), and helix angle (HA) maps were created using a cDTI post-processing platform developed in-house. The mean ± SD global FA was 0.613 ± 0.044, MD was 0.750 ±0.154 × 10^-3^ mm^2^/s and HA was epicardium -34.3 ± 7.6°, mesocardium 3.5 ± 6.9° and endocardium 38.9 ± 8.1°. Comparison of initial and repeat studies showed global interstudy reproducibility for FA (SD= ± 0.045, Coefficient of Variation (CoV) = 7.2%), MD (SD = ± 0.135 × 10–3 mm^2^/s, CoV = 18.6%) and HA (epicardium SD = ± 4.8°; mesocardium SD = ± 3.4°; endocardium SD = ± 2.9°). Reproducibility of FA was superior to MD (p = 0.003). Global MD was significantly higher in the septum than the reference lateral wall (0.784 ± 0.188 vs 0.750 ± 0.154 ×10^-3^mm^2^/s, p < 0.001). Septal HA was significantly lower than the reference lateral wall in all 3 transmural layers (from -8.3° to -10.4°, all p < 0.001). This is the first study to assess the interstudy reproducibility of DTI in the human HCM heart in-vivo and the largest cDTI study in HCM to date. The results showed good reproducibility of FA, MD and HA which indicates that current technology yields robust in-vivo measurements that have potential clinical value. The interpretation of regional differences in the septum requires further investigation.

### Fiber architecture in remodeled myocardium revealed with a quantitative diffusion CMR tractography framework and histological validation

The aim of this study was therefore to develop a technique for quantitative 3D diffusion CMR tractography of the heart, and to apply this method to quantify fiber architecture in the remote zone of remodeled hearts [26]. Diffusion Tensor CMR of normal human, sheep, and rat hearts, as well as infarcted sheep hearts was performed ex vivo. In normal hearts, the subendocardial and subepicardial myofibers had a positive and negative HA, respectively, forming a symmetric distribution around the midmyocardium. However, in the remote zone of the infarcted hearts, a significant positive shift in HA was observed. The ratio between negative and positive HA variance was reduced from 0.96 ± 0.16 in normal hearts to 0.22 < 0.05 in the remote zone of the remodeled hearts (p < 0.05). This was confirmed histologically by the reduction of HA in the subepicardium from -52.03° ± 2.94° in normal hearts to -37.48° ± 4.05° in the remote zone of the remodeled hearts (p < 0.05). The authors conclude that there is a significant reorganization of the 3D fiber continuum in the remote zone of remodeled hearts. The positive (rightward) shift in HA in the remote zone is greatest in the subepicardium, but involves all layers of the myocardium. Tractography-based quantification in remodeled hearts may provide a framework for assessing regional changes in the left ventricle following infarction.

## Congenital heart disease

CMR of congenital heart disease has become relatively mature, although quality interpretation requires training. There has been interest in assessing ventricular mechanics in congenital disease [27], comparing CMR volumetry with echo [28], defining clinical protocols [29], defining CMR in congenital disease in children [30], and pregnant women [31], and improving the understanding pathophysiology in these complex patients [32-34]. The use of 4D flow velocity data allows the visualisation of large scale vorticity and the retrospective measurement of flow in vessels within the volume covered.

### 4D cardiovascular magnetic resonance velocity mapping of alterations of right heart flow patterns and main pulmonary artery hemodynamics in tetralogy of Fallot

This study using 4D CMR velocity acquisitions studied flow through the right heart of patients with repaired tetralogy of Fallot (rTOF) compared with healthy volunteers [35]. This technique allows the time courses and large scale topologies of flow through the cavities to be compared. Right atrial filling predominantly in diastole in patients as opposed to systole in volunteers and abnormal vortical flow patterns in each right heart cavity in rTOF were reported. As in other 4D flow studies, effective averaging of velocities for each cardiac phase from data acquired over multiple cycles is a limitation, and the likely presence of multiple variables between individual patients such as those of cavity geometry, contractile function and valve function, make thorough and meaningful comparative analysis challenging.

### Systemic-to-pulmonary collateral flow in patients with palliated univentricular heart physiology: measurement using cardiovascular magnetic resonance 4D velocity acquisition

In this study, whole-heart 4D velocity acquisitions as well as five 2D flow acquisitions per study were obtained in patients with single ventricle physiology, 14 with bidirectional cavopulmonary connection [BCPC], and 15 with Fontan connections [36]. Good agreement was found between shunt calculations from the two types of acquisition, with the 4D approach allowing slightly shorter acquisition times of about 12 rather than 17 minutes. Measurements of the amount of shunting and the distributions of flow between the two lungs and between upper and lower body were documented.

### High-resolution motion compensated MRA in patients with congenital heart disease using extracellular contrast agent at 3 Tesla

In this study, ECG- and navigator-gated high-resolution MRA sequence (HR MRA) with slow infusion of extracellular contrast agent was implemented at 3 Tesla for the assessment of congenital heart disease and compared to standard first-pass MRA (FP MRA) in 34 patients, median age 13 years, with congenital heart disease [37]. HR-MRA was found to deliver better image quality compared to FP-MRA and could be integrated into a standard CMR-protocol for patients with CHD without additional examination time.

## Flow

### Feasibility of quantification of the distribution of blood flow in the normal human fetal circulation using CMR: a cross-sectional study

In this study, CMR phase contrast measurements of flow were obtained in key parts of circulations of 12 late gestation human foetuses during late pregnancy [38]. As no ECG suitable for fetal CMR gating was obtainable, a novel retrospective gating technique known as metric optimised gating was used. Measurements of flow recorded included: combined ventricular output, main pulmonary artery, ascending aorta, superior vena cava, ductus arteriosus, pulmonary branches, descending aorta, umbilical vein and foramen ovale. These offer novel non-invasive information on human prenatal circulatory physiology.

### A multi-center inter-manufacturer study of the temporal stability of phase-contrast velocity mapping background offset errors

Measurements of volume flow by phase-contrast velocity acquisitions can be compromised by background or phase offset errors [39]. This study assessed the temporal stability of such errors in 3 different 1.5T CMR systems, GE Signa Excite, Philips Intera and Siemens Avanto, over 8 weeks [40]. Retrospectively-gated velocity was acquired with fixed parameters through a predetermined oblique plane, repeated 5 times in rapid succession each week. Temporal drift in the baseline offset was insignificant on two machines (0.3 cm/s, 0.2 cm/s), and marginally insignificant on the third machine (0.5 cm/s) due to an apparent heating effect. During a typical patient study, background drift was insignificant, but over a longer timescale of 8 weeks, insignificant drift (0.4 cm/s) occurred on one, with larger drifts (0.9 cm/s, 0.6 cm/s) on the other 2 systems. This implied that, while static phantom correction of errors after a scan and probably after a day of scanning are likely to be effective, phantom correction after several weeks delay, or corrective calculation based on pre-stored background offset data, are less likely to be reliable.

### Velocity encoded cardiovascular magnetic resonance to assess left atrial appendage emptying

The left atrial appendage (LAA) can be a source of thrombi that can cause embolic stroke, and impaired LAA function is thought to predispose to thrombosis. This study explored the feasibility of assessing LAA emptying by velocity encoded CMR, comparing it with trans esophageal echo (TEE) measures, in 18 patients with sinus rhythm and 12 with atrial fibrillation [41]. It was concluded that assessment of active and passive LAA emptying CMR was feasible although further evaluation would be required for potential applications such as risk stratification.

### A non-invasive clinical application of wave intensity analysis based on ultrahigh temporal resolution phase-contrast cardiovascular magnetic resonance

Wave intensity analysis, traditionally derived from invasively acquired pressure and velocity data, were here derived from non-invasive CMR measures of velocity and area from a high-resolution breath hold spiral phase-contrast acquisition. Ascending and descending aortic flows were recorded in healthy adult volunteers and compared with results from 15 patients with coronary heart disease [42]. Patients had higher wave speed, and lower forward compression wave and forward expansion wave peaks, which may be attributable to their older age and reduced aortic compliance as much as to compromised ventricular function. The semi-automated analyses were found to be reproducible between observers, although inter-study reproducibility was not assessed. The approach shows potential for future non-invasive investigation of wave intensity in large vessels.

### Cardiac output and cardiac index measured with cardiovascular magnetic resonance in healthy subjects, elite athletes and patients with congestive heart failure

This study used velocity encoded CMR to establish whether cardiac index (CI, cardiac output indexed to body surface area) decreases with age in healthy volunteers and compared the CI values of athletes and patients with congestive heart failure (CHF) [43]. CI was found to decrease with age, without significant differences between males and females in the cohort of 144 volunteers studied. No differences of CI were found between 60 athletes and the volunteers at rest, but CI was lower in 157 patients with congestive heart failure.

## Aorta

CMR is widely used for assessment of the aorta in both congenital and acquired conditions and is particularly well suited for functional of compliance [44], and longitudinal follow-up of aortic dimensions [45]. These papers continue to expand the value of CMR in this major artery.

### Bramwell-hill modeling for local aortic pulse wave velocity estimation: a validation study with velocity-encoded cardiovascular magnetic resonance and invasive pressure assessment

The Bramwell-Hill model describes the relation between vascular wall stiffness expressed in aortic distensibility and the pulse wave velocity (PWV) [46]. In this study the authors showed that CMR with velocity-encoding is the optimal approach for studying Bramwell-Hill associations between local PWV and aortic distensibility [47]. This approach enables non-invasive estimation of local pulse pressure and distensibility.

### Normal values of aortic dimensions, distensibility, and pulse wave velocity in children and young adults: a cross-sectional study

This study provides normal CMR values for cross-sectional areas, distensibility and pulse wave velocity of the thoracic aorta in children and young adolescents and may serve as a reference for the detection of pathological changes of the aorta [48].

### Evaluation of 3D blood flow patterns and wall shear stress in the normal and dilated thoracic aorta using flow-sensitive 4D CMR

CMR is being used increasingly to investigate 4D flow patterns in the heart and great vessels [49]. In this study flow and vessel wall parameters were investigated in patients with dilated ascending aorta, age-matched subjects, and healthy volunteers. Increase in ascending aorta diameter is significantly correlated with the presence and strength of supra-physiologic-helix and vortex formation in the ascending aorta, as well with decrease in systolic regional wall shear stress and increase and oscillatory shear index [50].

### T1 mapping & extracellular volume

T1 mapping is the technique of the moment, and there has been a large expansion in papers since 2011 published on this subject in JCMR [51,52] and other literature this year.

### Extracellular volume fraction mapping in the myocardium, part 1: evaluation of an automated method

This manuscript describes the development and evaluation of a method of pixel-wise mapping of extracellular volume (ECV) fraction [53]. The method incorporates a novel approach to motion correction of a series of inversion recovery images with widely varying contrasts that is used for the T1-mapping, along with a new scheme for co-registration of the pre-and post-contrast T1-maps. The methods were demonstrated to significantly improve the image quality of ECV maps and the described implementation which is fully automated could be simply incorporated into a clinical workflow.

### Extracellular volume fraction mapping in the myocardium, part 2: initial clinical experience

As a follow up to “Extracellular volume fraction mapping in the myocardium, part 1: evaluation of an automated method" [53], the purpose of Part II was to provide the “Initial Clinical Experience” and to show how this method has been feasible in patients, and then to provide initial clinical results [54]. The authors hypothesized that quantitative assessment of myocardial ECV would be clinically useful for detecting both focal and diffuse myocardial abnormalities in a variety of common and uncommon heart diseases. In this preliminary study they used the method on a total of 156 subjects including 62 with normal findings, 33 patients with chronic myocardial infarction (MI), 33 with hypertrophic cardiomyopathy (HCM), 15 with non-ischemic dilated cardiomyopathy (DCM), 7 with acute myocarditis, 4 with cardiac amyloidosis, and 2 with systemic capillary leak syndrome (SCLS). Although, due to small numbers, they cannot prove their hypothesis in this study they do set the ground work for future larger scale methods using these improved methods.

### T1 mapping of the myocardium: Intra-individual assessment of the effect of field strength, cardiac cycle and variation by myocardial region

This work investigates the intra-individual variations in T1 mapping and extracellular volume at different field strengths, myocardial regions and cardiac phases [55]. The paper probes these important and relevant issues relating to T1 mapping. The authors’ conclude that when using the same contrast at equimolar dose, ECV does not vary with field strength. They also suggest that the MOLLI measurement shows small changes in myocardial blood volume dependant on the measurement time within the cardiac cycle. Additionally, minor but statistically significant differences in ECV between myocardial regions were detected but absolute differences were small and depending on the application may well not be relevant.

T1 mapping of the myocardium: intra-individual assessment of post-contrast T1 time evolution and extracellular volume fraction at 3T for Gd-DTPA and Gd-BOPTA.

This manuscript focuses on the impact of the contrast agent in the determination of normal ECV and T1 values for healthy volunteers and also assesses stability of ECV over time [56]. The results showed a minor but significant difference with contrast agent of 2% between Gd-DTPA and Gd-BOPTA. The authors’ describe how the binding of Gd-BOPTA to human serum albumin will result in a lower molecular tumbling rate of the molecule and a longer rotational MR correlation time leading to an increased relaxivity. Also that, since albumin is mainly present in blood, the distribution between blood and myocardium is expected to be different between Gd-BOPTA and Gd-DTPA. They believe that this should affect the ratio of the change in relaxation rate of myocardium and blood and could explain the difference in ECV values. The authors also conclude that the small but significant linear increase in ECV with time after a bolus of contrast will necessitate either careful attention to imaging delay time or post-hoc correction.

### Comparison of T1 mapping techniques for ECV quantification. histological validation and reproducibility of ShMOLLI versus multibreath-hold T1 quantification equilibrium contrast CMR

In this work, the authors’ introduced the use of a single breath-hold T1 mapping technique (ShMOLLI) for the equilibrium contrast method they had initially described and validated for the measurement of ECV [57]. The results were compared to the original multi breath-hold IR technique that they had used previously in healthy volunteers and patients with HCM, severe aortic stenosis and amyloid. The study found that all subjects completed ShMOLLI, whilst a few could not fully complete the multi breath-hold T1 measurements and there was good correlation between results from the two techniques, with ShMOLLI achieving slightly higher correlation with histology.

## Interventional CMR

### Direct cooling of the catheter tip increases safety for CMR-guided electrophysiological procedures

The authors used an EP catheter’s own irrigation system for cooled-tip ablation and assessed the use of this to reduce the temperature at the blood-tissue interface [58]. Initially, in this phantom study the heating was measured at the catheter tip during typical sequence RF field exposure in the MR machine. Then using a sophisticated phantom with temperature sensors located at different distances from the surface, the ability of the catheter’s own irrigation system to prevent heating was tested. The authors’ concluded that the irrigated tip system could be used to increase MR safety of EP catheters by suppressing the effects of unwanted passive catheter heating due to RF exposure from the MR scanner.

### Remote control catheter navigation: options for guidance under MRI

In this review article a presentation is made of the various strategies which have been introduced for remotely steering vascular catheters within the MRI environment [59]. Following an introduction covering the potential advantages and challenges the various methods of catheter location and guidance are reviewed. Remote control catheter guidance systems were then compared and contrasted with respect to visualization, safety, and performance. The work concluded that the field was still in its infancy and that additional experimental studies will be needed prior to their use in humans.

### MRI active guidewire with an embedded temperature probe and providing a distinct tip signal to enhance clinical safety

In this study the emphasis was again aimed at safety. The authors’ describe a guide-wire design based on a loopless antenna [60]. Importantly, they modified this design by the incorporation a temperature sensor and a solenoid coil at the tip and then tested the performance in phantom and in vivo experiments. The detector design provided good tip visibility for the purpose of device tracking, and the temperature probe a welcome safety addition. The authors’ conclude that with this design we need not rely on prediction to ensure safe clinical operation as future implementations may modulate specific absorption rate (SAR) based on temperature feedback.

### Towards real-time cardiovascular magnetic resonance guided transarterial core valve implantation: in vivo evaluation in swine

The value of CMR in transarterial aortic valve implantation (TAVI) is under exploration [61,62]. In this pre-clinical study of MRI guided TAVI, the procedure was performed in 8 swine using the original CoreValve prosthesis and a modified, CMR-compatible delivery catheter without ferromagnetic components [63]. Due to the recent success of TAVI the topic is timely and the study illustrates nicely the technical progress in delivery systems, devices and real time (rt) CMR imaging techniques. Owing to the unlimited scan plane orientation and an unsurpassed soft-tissue contrast with simultaneous device visualization, rtCMR is presumed to allow safe device navigation and to offer optimal orientation for precise axial positioning. This work demonstrated the feasibility of rtCMR-guided TAVI using the nitinol-based CoreValve bioprosthesis.

## Cardiomyopathy

The phenotyping of cardiomyopathy is a major success story for CMR, and has led to gene discovery [64], and identification of subtle myocardial mechanical derangement [65]. CMR has made recent advances in unusual conditions such as wet beri-beri [66], takotsubo cardiomyopathy [67], lamin A/C dilated cardiomyopathy [68], limb girdle muscular dystrophy [69], Duchenne muscular dystrophy [70-72].

### The diagnosis of hypertrophic cardiomyopathy by cardiovascular magnetic resonance

Hypertrophic cardiomyopathy (HCM) is characterised by asymmetric hypertrophy, increased fibrosis and myocyte disarray. It represents the most common genetic disorder of the myocardium. There is a wide range of clinical expression, ranging from asymptomatic mutation carriers [73], to sudden cardiac death as the first manifestation of the disease. In spite of advances in genotyping, the spectrum of phenotype is wide. CMR is increasingly used to characterize morphologic, functional and tissue abnormalities associated with HCM. CMR appears to be highly relevant in the clinical as well as research evaluation of patients with overt as well as pre-clinical HCM. Late enhancement after gadolinium administration allows tissue characterization of myocardial fibrosis. It is present in approximately 60% of HCM patients with LVH. The method may potentially identify HCM patients at greatest risk for adverse cardiac events. CMR evaluation of HCM mutation carriers in an early stage of disease has yet to be extensively evaluated, but represents a promising method for exploring the inter-relationship between functional, morphologic and tissue abnormalities in HCM. This review assesses the role of CMR in characterising the current and future roles of CMR in HCM [74].

### Clinical utility of cardiovascular magnetic resonance in hypertrophic cardiomyopathy

In this review article, Maron considers the incremental role of CMR in patients with HCM [75]. Complementing the separate review by Radwa et al., in this article the role of CMR in high-risk HCM patient subgroups is discussed including those with thin-walled scarred LV apical aneurysms, end-stage systolic dysfunction, and massive LV hypertrophy. CMR observations to include thickening of the right ventricular wall as well as substantial morphologic diversity with regard to papillary muscles and mitral valve are discussed. The implications for management strategies in patients undergoing invasive septal reduction therapy is considered. Among HCM family members, CMR has identified unique phenotypic markers of affected genetic status in the absence of LV hypertrophy including: myocardial crypts, elongated mitral valve leaflets and late gadolinium enhancement. Understanding the prognostic significance of fibrosis is assessed. The clinical role of contemporary CMR in management is considered in this timely review article.

### Diagnostic and prognostic value of cardiovascular magnetic resonance in non-ischaemic cardiomyopathies

In this review, the current evidence base to support the role of CMR in the evaluation of cardiomyopathies is discussed [76]. The unique strength of CMR relates to the range of tissue characterisation and sequences available. Applications of this are well established in the diagnosis of cardiomyopathies and increasingly in directing therapy and guiding prognostic evaluation.

### Diffuse myocardial fibrosis in hypertrophic cardiomyopathy can be identified by cardiovascular magnetic resonance, and is associated with left ventricular diastolic dysfunction

This study was undertaken to detect and quantify diffuse myocardial fibrosis HCM patients using a histologically validated CMR post-contrast myocardial T1 mapping technique [77]. Furthermore, they investigated the relationships of both patterns of fibrosis to LV diastolic performance and clinical manifestations. This study demonstrated that patients with HCM have reduced post-contrast myocardial T1 times, consistent with the presence of diffuse interstitial fibrosis. Furthermore, the independent association of post-contrast myocardial T1 time with estimated LV filling pressure (E/e’) suggests a mechanistic link between altered myocardial composition and function. The non-invasive detection of diffuse fibrosis, in combination with standard LGE sequences to identify dense regional fibrosis, could allow a detailed evaluation of patterns of fibrosis in HCM. Further research utilizing this technique may enhance understanding of the relationships between HCM genetic mutations, abnormal myocardial structure and function, and risk stratification and may facilitate the future development of disease-modifying therapies.

### Myocardial scarring on cardiovascular magnetic resonance in asymptomatic or minimally symptomatic patients with “pure” apical hypertrophic cardiomyopathy

In this study, the authors found that myocardial fibrosis is seen in about 75% of patients with asymptomatic or mildly symptomatic ‘pure’ apical HCM patients [78]. They observed that apical segments, including apical cap, were the most frequently involved LGE sites in apical HCM patients. LGE was not limited to hypertrophic apical segments, and that it was also present in basal segments, at the anterior and posterior junctions between the septum and RV free wall, and additionally in non-hypertrophied segments of apical HCM. Importantly, LGE observed at apex (including apical cap) 48% of 97 apical segments showing LGE was in a “subendocardial” pattern, quite unlike the patchy or focal mid-wall LGE pattern frequently observed in asymmetrical septal HCM patients. Given that “ischemic-type” LGE is usually characterized by subendocardial involvement with or without extension into subepicardium, the subendocardial LGE pattern at apex in their population suggests previous subendocardial ischemia. This “ischemic-type” LGE is likely to be attributable to mechanisms other than epicardial coronary stenosis. A hypertrophied apical wall with relatively deficient coronary blood flow may be a plausible explanation for this “ischemic-type” LGE. However, an increment in intra-apical cavitary pressure that might subsequently lead to suboptimal perfusion to the subendocardial area could explain “ischemic-type” LGE at the apex, as well. It is unclear if these patterns are representative of adverse prognosis in this asymptomatic or minimally symptomatic apical HCM population, especially in the short term.

### The protein binding substance ibuprofen does not affect the T1 time or partition coefficient in contrast-enhanced cardiovascular magnetic resonance

In this study, the authors assessed the effect of co-medication with a typical protein binding drug (Ibuprofen) on contrast enhanced T1 mapping [79]. The authors consider the most important findings can be summarized as follows: 1.) Ibuprofen did not significantly affect T1 times of myocardium and blood when using a gadolinium based contrast agent with protein binding capacity (Gd-BOPTA) 2.) Correlations of T1 times of myocardium and blood and partition coefficient between exam 1 and 2 were excellent reflecting good reproducibility.

### Structural and functional cardiac changes in myotonic dystrophy type 1: a cardiovascular magnetic resonance study

Myotonic dystrophy type 1 (MD1) is a neuromuscular disorder with potential involvement of the heart and increased risk of sudden death. It is an autosomal dominant inherited disorder caused by an unstable expansion of a repetitive trinucleotide sequence (CTG) on chromosome 19. The prevalence varies from 2.1-14.3 per 100 000. MD1 is characterized by slowly progressive weakness of skeletal muscles, myotonia and involvement of several organ systems. An earlier age of onset and increased severity of clinical symptoms has been observed in subsequent generations and is related to degree of CTG expansion. Patients with MD1 usually die from respiratory or cardiac complications. Sudden death is considered to be the result of atrioventricular block or ventricular arrhythmias. Recent studies showed that severe electrocardiographic (ECG) abnormalities and atrial arrhythmias are independent risk factors, although with moderate sensitivity, for sudden death in MD1 patients. Although death from progressive heart failure is uncommon in patients with MD1 compared to other muscular dystrophies, left ventricular systolic dysfunction is associated with an increased risk of overall mortality and sudden death. Therefore, the picture emerges that MD1 patients have a complex cardiac phenotype including both the myocardium and the conduction system. The principal finding of this study is that structural and functional myocardial abnormalities are frequent in MD1 patients [80]. The presence of mild to moderate left ventricular systolic dysfunction, ventricular dilatation, myocardial hypertrophy or fibrosis was strongly associated with electrocardiographic conduction abnormalities. However, 16% of patients with a normal ECG still had myocardial alterations. These findings lend support to the concept that the myocardium is generally involved in the pathogenic process of MD1.

### Diffuse myocardial fibrosis evaluation using cardiac magnetic resonance T1 mapping: sample size considerations for clinical trials

The aim of this study was to determine the reproducibility and sample size of CMR fibrosis measurements that would be applicable in clinical trials [81]. Mean ECV and λ were both significantly higher in HF subjects than healthy (ECV: 0.287 ± 0.034 vs. 0.267 ± 0.028, p = 0.002; λ: 0.481 ± 0.052 vs. 442 ± 0.037, p < 0.001, respectively). The inter-study ECV and λ variation were about 2.8 times greater than the intra-study ECV and λ variation in healthy subjects (ECV: 0.017 vs. 0.006, λ: 0.025 vs. 0.009, respectively). The estimated sample size to detect ECV change of 0.038 or λ change of 0.063 (corresponding to ~3% increase of histological myocardial fibrosis) with a power of 80% and an alpha error of 0.05 for heart failure subjects using a two group design was 27 in each group, respectively. ECV and partition coefficient have a relatively low variability for repeat scans, and could be a viable tool for evaluating clinical trial outcome. Sample size estimation showed that a study with 27 participants in each group could detect a 0.038 change in ECV or 0.063 change in partition coefficient with 80% of power, which corresponding to about 3% increase in histological collagen tissue. ECV and λ quantification have a low variability across scans, and could be a viable tool for evaluating clinical trial outcome.

### The impact of repeated marathon running on cardiovascular function in the aging population

Participation in strenuous aerobic physical activity is increasing among the aging population. The cardiovascular effects of acute strenuous exercise, specifically marathon running, have demonstrated a transient increase in cardiac biomarkers and right ventricular (RV) systolic dysfunction using multimodality cardiac imaging, including transthoracic echocardiography (TTE) and cardiovascular magnetic resonance (CMR). Little is known, however, on the cardiovascular effects of repeated marathon running in individuals over the age of 50. The aims of the current study were two-fold: 1) To assess the extent and severity of cardiac dysfunction after the completion of full marathon running in elite individuals >50 years of age using cardiac biomarkers, TTE and CMR; and 2) If there is evidence of LGE on CMR, to detect the presence of silent coronary artery disease using cardiac computed tomography (CCT) [82]. Marathon running in individuals over the age of 50 is associated with a transient, yet reversible increase in cardiac biomarkers and RV systolic dysfunction. The presence of myocardial fibrosis in older marathon athletes is infrequent, but when present, may be due to underlying occult coronary artery disease. Larger studies are needed to confirm these findings.

### Effects of combined deferiprone with deferoxamine on right ventricular function in thalassaemia major

With the development of the T2* technique [83], and the black-blood sequence [84], CMR has provided new insights into iron-overload cardiomyopathy, as the myocardial iron concentration [85], prognosis [86], and its toxic effect on ventricular function [87], can be assessed at the same time with the same high-fidelity technique. Recent advances include identification of the lack of myocardial fibrosis [88], the involvement of the right ventricle [89], early strain abnormalities [90], and improved prognosis [91]. Combination therapy with deferoxamine and oral deferiprone is superior to deferoxamine alone in removing cardiac iron and improving left ventricular ejection fraction (LVEF). The right ventricle (RV) is also affected by the toxic effects of iron and may cause additional cardiovascular perturbation. In this study, the authors assessed the effects of combination therapy on the RV in thalassaemia major (TM) using CMR [92]. In the RCT, combination therapy with deferoxamine and deferiprone was superior to deferoxamine alone for improving RVEF (3.6 vs 0.7%, p = 0.02). The increase in RVEF was greater with lower baseline T2* 8–12 ms (4.7 vs 0.5%, p = 0.01) than with T2* 12–20 ms (2.2 vs 0.8%, p = 0.47). In patients with severe cardiac siderosis, substantial improvement in RVEF was seen with open-label combination therapy (10.5% ± 5.6%, p < 0.01). Adding deferiprone to deferoxamine has beneficial effects on both RV and LV function in TM patients with cardiac siderosis.

### Cardiovascular magnetic resonance findings in a pediatric population with isolated left ventricular non-compaction

Several echocardiographic and CMR measurements have been proposed to diagnose LVNC. There has been an association between adult patients with isolated LVNC and depressed left ventricular function; however the reason for this remains unclear. In adults, it has been found that some of these measurements such as NC/C ratio and NCMA are well correlated with systolic dysfunction. However, in children these relations have not been yet established. In this study the authors investigated the relation between different anatomical measurements with ventricular performance using CMR in a pediatric population [93]. They found that only five patients had a decreased EF (< 53%) and that there was a poor correlation between EF and measurements such as CMM, CMA, NC/C ratio and the X:Y ratio proposed by Chin et al. A better correlation was found between EF and NCMA (R = 0.67). In this study the NCMA was calculated as the sum of the NCMA on LA and 4Ch views; this was done to include any septal and lateral walls that may involve any degree of non-compaction. The distribution of non-compaction in children was similar to published adult data with a predilection for apical, mid-inferior and mid-lateral segments. Five patients had systolic dysfunction with decreased EF. The number of affected segments was the strongest predictor of systolic dysfunction, all five patients had greater than 9 affected segments. Basal segments were less commonly affected but they were affected only in these five severe cases. The segmental pattern of involvement of non-compaction in children is similar to that seen in adults. Systolic dysfunction in children is closely related to the number of affected segments.

## Valves

The application of CMR to the assessment of valvular heart disease continues to increase, notably with respect to aortic stenosis [94]. This is in part due to greater appreciation of its capability, wider accessibility, and an expanding scientific literature.

### Heart valve disease: investigation by cardiovascular magnetic resonance

This excellent review summarises and discusses the additive value of CMR in the investigation of valve disease, highlighting not only its many advantages as an adjunct to echocardiography, but addressing also the limitations of the technique [95]. The ability to image in any plane, facilitating direct measurement of valve area, and the ability to accurately quantify flow are strengths of this technique. In combination with the unparalleled and established accuracy of CMR in quantifying ventricular volumes and mass, the technique offers evaluation not only of the valve, but also the sequelae of the disease on the heart. This is not withstanding the additional information offered by the administration of Gadolinium contrast. Acknowledgment is made of the limitations imposed by image slice thickness with respect to partial volume effects, the potential underestimation of aortic regurgitation due to through plane motion of the aortic root, and also the limited ability to identify small highly mobile objects as a consequence of acquiring images over several cardiac cycles. The review considers the optimal imaging of each valve and emphasises the increasingly prominent role of CMR in investigating the pulmonary valve and right ventricle.

### Left ventricular remodeling and hypertrophy in patients with aortic stenosis: insights from cardiovascular magnetic resonance

This manuscript describes the novel application of CMR quantification of indexed left ventricular volumes and mass index to investigate and characterise the impact of the pressure loading in aortic stenosis on left ventricular remodelling and hypertrophy in 91 patients with echocardiographic moderate or severe aortic stenosis [96]. There was no significant correlation between the degree of hypertrophy (r^2^ = 0.012, P = 043), adaptive response of the ventricle (P = 0.22) and the severity of aortic stenosis as assessed by CMR. Although concentric hypertrophy was the most commonly observed response (n = 34, 37% of patients), six patterns of LV adaptation were observed. Asymmetric patterns of wall thickening were frequently noted (n = 14) with some phenotypic overlap with hypertrophic cardiomyopathy.

### Cardiovascular magnetic resonance evaluation of aortic stenosis severity using single plane measurement of effective orifice area

Further consideration of the utility of CMR in the assessment of aortic stenosis is made in this study seeking to validate novel CMR estimation of valve effective orifice area (EOA) by a single plane method using jet shear layer detection (JSLD) against CMR estimation of EOA by the continuity equation and the customary echocardiographic measure of EOA by the continuity equation [97]. JSLD and the simplified JSLD method assessed are based on the acoustical source term (AST) concept which interrogates the source of the acoustic noise created by the accelerated flow beyond the stenotic valve. In vitro assessment of the CMR technique agreed well with theoretical prediction, in addition to in vivo study in 8 healthy volunteers and 37 patients across the spectrum of severity of aortic stenosis. CMR estimation of EOA by both the novel methods (r = 0.93) and continuity equation (r = 0.88) all agreed well with EOA estimation by TTE, supporting use of CMR, especially where echocardiography is suboptimal or inconclusive. Reproducibility was favourable for CMR compared to echocardiography regardless of method.

## Myocardial perfusion

Although myocardial perfusion CMR has been possible for many years, the clinical sequence and data processing remain variable between centres. The papers below help to close the gap between research and clinical practice including reports from the large MR-INFORM and the MR-IMPACT trials. Recent developments in accelerated acquisitions, quantification perfusion acquisition techniques [98], and even new contrast agents [99], will feed into clinical practice in due course.

### Design and rationale of the MR-INFORM study: stress perfusion cardiovascular magnetic resonance imaging to guide the management of patients with stable coronary artery disease

In patients with stable coronary artery disease (CAD), decisions regarding revascularisation are primarily driven by the severity and extent of coronary luminal stenoses as determined by invasive coronary angiography. More recently, revascularisation decisions based on invasive fractional flow reserve (FFR) have shown improved event free survival. Perfusion CMR has been shown to be non-inferior to nuclear perfusion imaging in a multi-centre setting and superior in a single centre trial. In addition, it is similar to invasively determined FFR and therefore has the potential to become the non-invasive test of choice to determine need for revascularisation. This paper describes the rationale and study protocol for the MR-INFORM trial comparing MR and FFR [100]. The MR-INFORM study is a prospective, multi-centre, randomised controlled non-inferiority, outcome trial. The objective is to compare the efficacy of two investigative strategies for the management of patients with suspected CAD. Patients presenting with stable angina are randomised into two groups: 1) The FFR-INFORMED group has subsequent management decisions guided by coronary angiography and fractional flow reserve measurements. 2) The MR-INFORMED group has decisions guided by stress perfusion CMR. The primary end-point will be the occurrence of major adverse cardiac events (death, myocardial infarction and repeat revascularisation) at one year. MR INFORM will assess whether an initial strategy of perfusion CMR is non-inferior to invasive angiography supplemented by FFR measurements to guide the management of patients with stable coronary artery disease. Non-inferiority of perfusion CMR to the current invasive reference standard of FFR would establish perfusion CMR as an attractive non-invasive alternative to current diagnostic pathways.

Superior diagnostic performance of perfusion-cardiovascular magnetic resonance versus SPECT to detect coronary artery disease: The secondary endpoints of the multicenter multivendor MR-IMPACT II (Magnetic Resonance Imaging for Myocardial Perfusion Assessment in Coronary Artery Disease Trial).

Perfusion-cardiovascular magnetic resonance (CMR) is generally accepted as an alternative to SPECT to assess myocardial ischemia non-invasively. However its performance vs gated-SPECT and in sub-populations is not fully established. In this multicenter study, the diagnostic performance of perfusion-CMR and gated-SPECT was compared for the detection of CAD in various populations using conventional x-ray coronary angiography (CXA) as the standard of reference [101]. In 33 US and Europe centers, 533 patients were enrolled in this multivendor trial. SPECT and CXA were performed within 4 weeks before or after CMR in all patients. Prevalence of CAD in the sample was 49% and 515 patients received MR contrast medium. Drop-out rates for CMR and SPECT were 5.6% and 3.7%, respectively (ns). The study was powered for the primary endpoint of non-inferiority of CMR vs SPECT for both, sensitivity and specificity for the detection of CAD (using a single-threshold reading). The results for the primary endpoint were reported elsewhere, and in this article the secondary endpoints of diagnostic performance of CMR versus SPECT in subpopulations such as multi-vessel disease (MVD), in men, in women, and in patients without prior myocardial infarction (MI) are presented. The diagnostic performance (= area under ROC = AUC) of CMR was superior to SPECT (p = 0.0004, n = 425) and to gated-SPECT (p = 0.018, n = 253). CMR performed better than SPECT in MVD (p = 0.003 vs all SPECT, p = 0.04 vs gated-SPECT), in men (p = 0.004, n = 313) and in women (p = 0.03, n = 112) as well as in the non-infarct patients (p = 0.005, n = 186 in 1–3 vessel disease and p = 0.015, n = 140 in MVD). The authors conclude that the diagnostic performance of perfusion-CMR to detect CAD was superior to perfusion SPECT in the entire population and in sub-groups. Perfusion-CMR can be recommended as an alternative for SPECT.

### Comparison of exercise electrocardiography and stress perfusion CMR for the detection of coronary artery disease in women

Exercise electrocardiography (ECG) is frequently used in the work-up of patients with suspected coronary artery disease (CAD), however the accuracy is reduced in women. CMR stress testing can accurately diagnose CAD in women. This study reports a direct comparison of CMR to ECG [102]. The authors prospectively enrolled 88 consecutive women with chest pain or other symptoms suggestive of CAD. Patients underwent a comprehensive clinical evaluation, exercise ECG, perfusion CMR and infarct imaging, and x-ray coronary angiography (CA) within 24 hours. CAD was defined as stenosis ≥70% on quantitative analysis of CA. Exercise ECG, CMR and CA was completed in 68 females (age 66.4 ± 8.8 years, number of CAD risk factors 3.5 ± 1.4). The prevalence of CAD on CA was 29%. The Duke treadmill score (DTS) in the entire group was -3.0 ± 5.4 and was similar in those with and without CAD (-4.5 ± 5.8 and -2.4 ± 5.1; P = 0.12). Sensitivity, specificity and accuracy for CAD diagnosis was higher for CMR compared with exercise ECG (sensitivities 85% and 50%, P = 0.02, specificities 94% and 73%, P = 0.01, and accuracies 91% and 66%, P = 0.0007, respectively). Even after applying the DTS the accuracy of CMR was higher compared to exercise ECG (area under ROC curve 0.94 ± 0.03 vs 0.56 ± 0.07; P = 0.0001). The authors conclude that in women with intermediate-to-high risk for CAD who are able to exercise and have interpretable resting ECG, perfusion CMR has higher accuracy for the detection of relevant obstruction of the epicardial coronaries than the exercise ECG.

### Considerations when measuring myocardial perfusion reserve by cardiovascular magnetic resonance using regadenoson

Adenosine perfusion CMR can quantify myocardial perfusion reserve. While regadenoson is increasingly employed due to ease of use, imaging protocols have not been standardized. The authors sought to determine the optimal regadenoson CMR protocol for quantifying myocardial perfusion reserve index (MPRi), and whether regadenoson stress imaging should be performed before or after rest imaging [103]. Twenty healthy subjects underwent CMR perfusion imaging during resting conditions, during regadenoson-induced hyperemia (0.4 mg), and after 15 min of recovery. In 10/20 subjects, recovery was facilitated with aminophylline (125 mg). Myocardial time-intensity curves were used to obtain left ventricular cavity-normalized myocardial up-slopes. MPRi was calculated in two different ways: as the up-slope ratio of stress to rest (MPRi-rest), and the up-slope ratio of stress to recovery (MPRi-recov). In all 20 subjects, MPRi-rest was 1.78 ± 0.60. Recovery up-slope did not return to resting levels, regardless of aminophylline use. Among patients not receiving aminophylline, MPRi-recov was 36 ± 16% lower than MPRi-rest (1.13 ± 0.38 vs. 1.82 ± 0.73, P = 0.001). In the 10 patients whose recovery was facilitated with aminophylline, MPRi-recov was 20 ± 24% lower than MPRi-rest (1.40 ± 0.35 vs. 1.73 ± 0.43, P = 0.04), indicating incomplete reversal. In 3 subjects not receiving aminophylline and 4 subjects receiving aminophylline, up-slope at recovery was greater than at stress, suggesting delayed maximal hyperemia. The authors conclude that MPRi measurements from regadenoson CMR are underestimated if recovery perfusion is used as a substitute for resting perfusion, even when recovery is facilitated with aminophylline.

### Myocardial first-pass perfusion imaging with hybrid-EPI: frequency-offsets and potential artefacts

First-pass myocardial perfusion is often imaged with a tailored hybrid centric interleaved echo-planar-imaging sequence, providing rapid image acquisition with good contrast enhancement. The centric interleaved phase-encode order minimises the effective time-of-echo but it is sensitive to frequency-offsets. This article aims to show possible artefacts that might originate with this sequence, in the context of first-pass perfusion imaging, when frequency-offsets are present [104]. Numerical and phantom simulations were used to illustrate the effects of frequency-offsets and non-uniform magnitude modulation with this sequence in a typical perfusion protocol. In vivo data was post-processed to analyse the h-EPI’s sensitivity to the frequency-offsets. The centric phase-order was shown to be highly sensitive to frequency-offsets due to its symmetrical phase slope. Resulting artefacts include blurring, and splitting of the image into two identical copies along the phase-encode direction. It was also shown that frequency-offsets can introduce signal loss and ghosting of the right ventricle signal into the myocardium. The in vivo results were confirmed by numerical and phantom simulations. Magnitude modulation effects were found to be small. The authors conclude that imaging first-pass myocardial perfusion with an hybrid centric echo-planar-imaging sequence can be corrupted with ghosting and splitting of the image due to frequency-offsets.

### Perfusion cardiovascular magnetic resonance: comparison of an advanced, high-resolution and a standard sequence

Technical advances in perfusion CMR, particularly accelerated data acquisition methods, allow myocardial perfusion imaging with unprecedented spatial resolution. However, it is not clear how implementation of these recent advances affects perfusion image quality, signal and contrast to noise ratios (SNR & CNR) and the occurrence of important artefacts in routine clinical imaging. The objective of this study was therefore to compare a standard and an advanced, high-resolution perfusion sequence [105]. A standard ultrafast gradient echo perfusion sequence (st-GrE) was compared with an advanced kt-accelerated steady state free precession sequence (ktBLAST-SSFP) at 1.5 T in healthy volunteers (n = 16) and in patients (n = 32) with known or suspected coronary artery disease. Volunteers were imaged with both sequences at rest and patients underwent stress and rest imaging with either st-GrE or ktBLAST-SSFP prior to X-ray coronary angiography. In normal hearts ktBLAST-SSFP imaging resulted in significantly improved image quality (p = 0.003), SNR (21.0 ± 6.7 vs. 18.8 ± 6.6; p = 0.009), CNR (15.4 ± 6.1 vs. 14.0 ± 6.0; p = 0.034) and a reduced extent (p = <0.0001) and transmurality (p = 0.0001) of DRA. In patients ktBLAST-SSFP imaging resulted in significantly improved image quality (p = 0.012), and a reduced extent (p = <0.0001), duration (p = 0.004) and transmurality (p = <0.0001) of DRA. Sensitivity and specificity for the detection of CAD against X-ray angiography was comparable with both sequences. There was a non-significant trend towards increased respiratory artefacts with ktBLAST-SSFP in both patients and volunteers. The authors conclude that advanced high resolution perfusion CMR using a k-t-accelerated SSFP technique results in significantly improved image quality, SNR and CNR and a reduction in the extent and transmurality of DRA compared to a standard sequence. These findings support the use of advanced perfusion sequences for clinical perfusion imaging however further studies exploring whether this results in improved diagnostic accuracy are required.

### Quantitative myocardial perfusion in mice based on the signal intensity of flow sensitized CMR

In the conventional approach to arterial spin labeling in the rodent heart, the relative difference in the apparent T1 relaxation times corresponding to selective and non-selective inversion is related to perfusion via a two compartment model of tissue. But accurate determination of T1 in small animal hearts is difficult and prone to errors due to long scan times and high heart rates. In this study, the authors introduce the theoretical frame work for an alternative method based on the signal intensity of slice-select and non-select inversion recovery images at a single inversion time at short repetition time [106]. A modified Bloch equation was solved to derive perfusion as a function of signal intensity of flow sensitized segmented gradient echo acquisitions. A 2 compartment fast exchanging model of tissue was assumed. To test the new technique first it was implemented on a flow phantom and then it was compared with the conventional T1 method in an in vivo study of healthy C57BL/6 mice (n = 12). Finally the SI-method was used in comparison to a Late Gadolinium Enhanced (LGE) method to qualitatively and quantitatively assess perfusion deficits in an ischemia-reperfusion mouse model (n = 4). The myocardial perfusion of healthy mice obtained by the SI-method, 5.6 ± 0.5 ml/g/min, (mean ± standard deviation) was similar (p = 0.38) to that obtained by the conventional method, 5.6 ± 0.3 ml/g/min. The variance in perfusion within the left ventricle was less for the SI-method than that for the conventional method (p < 0.0001). The LGE regions of the ischemia reperfusion model were matched with regions of hypo-perfusion in the perfusion map. The average perfusion in the hypoperfused region among all 4 IR mice was 1.2 ± 0.9 ml/g/min and that of the remote region was 4.4 ± 1.2 ml/g/min. The proposed signal intensity based ASL method with a segmented acquisition scheme allows accurate high resolution perfusion mapping in small animals. It’s short scan time, high reproducibility and ease of post process makes it a robust alternative to the conventional ASL technique that relies on T1 measurements.

## Acute and chronic coronary artery disease

CMR is invaluable to the study of patients with ischaemic heart disease and has made a major impact on this field since the first reports of inversion-recovery techniques to delineate myocardial infarction. The use of CMR in the acute cardiac setting has been reviewed [107], and recent application has also been reported in improving understanding of the behaviour of the heart during ventricular fibrillation [108], and response to stem cell treatment [109]. In the acute setting, the value of myocardial perfusion has been reported [110,111], as well as assessment of salvage [112]. The role of CMR in the chronic setting of coronary disease has been extensively studied. The prevalence of infarction in heart failure [113], and data on the prediction of predicting revascularization improvement in function was recently reported [114]. In known or suspected coronary artery disease, there has been a review of dobutamine stress CMR [115], and data on the prognosis of myocardial scar in patients with normal wall motion [116], as well as a study of the role of ischemic preconditioning [117]. There have also been reports of new techniques in animals [118-120]. The papers below show how CMR can be used in a number of ways to characterize acute infarction.

### Prognostic value at 5 years of microvascular obstruction after acute myocardial infarction assessed by cardiovascular magnetic resonance

Microvascular obstruction (MVO) or the ‘no-reflow’ phenomenon is an established complication of coronary reperfusion therapy for acute myocardial infarction. It is increasingly recognized as a poor prognostic indicator and marker of subsequent adverse LV remodeling. Klug reported the relation between microvascular obstruction (MVO) on first-pass gadolinium images and outcomes in 129 patients treated with primary angioplasty for ST-segment elevation myocardial infarction (STEMI) [121]. At a median follow-up of 52 months, 63 pre-defined endpoints (a composite of death, myocardial re-infarction, stroke, repeat revascularization, recurrence of ischemic symptoms, atrial fibrillation, congestive heart failure and hospitalization) had occurred. The primary endpoint occurred in 66.2% of patients with and 42.4% of patients without MVO (p < 0.05). The presence of early MVO was associated with worse event-free survival and was the strongest independent predictor for the occurrence of the primary endpoint in a multivariable Cox regression analysis adjusting for age, ejection fraction and infarct size (hazard ratio: 2.79, 95%-CI 1.25-6.25, p = 0.012). Patients with early MVO also had larger infarcts and a lower EF. Although other studies have shown that MVO is a prognostic marker for clinical endpoints [122], the follow-up has previously only been relatively short-term and this study was able to confirm early MVO as an independent long-term prognosticator for morbidity after acute myocardial infarction.

### Assessment of intramyocardial hemorrhage by T1-weighted cardiovascular magnetic resonance in reperfused acute myocardial infarction

Due to their inherent susceptibility to field inhomogeneity, T2* sequences have been used to assess intramyocardial hemorrhage (IMH) [123]. The methemoglobin within the IMH contains causes shortening of all MR relaxation parameters in a similar way to myocardial iron overload. IMH represents severe reperfusion injury in acute myocardial infarction and has the potential to be used as a surrogate marker to assess the effects of preventive strategies to reduce this. Pedersen used T1-weighted inversion recovery, T2-STIR (short tau inversion recovery) and T2* sequences to assess myocardial damage in a porcine model of myocardial infarction [124]. The CMR images were compared with the area of IMH at histology. Despite relatively wide limits of agreement, the authors found that the T1 technique demonstrated a superior specificity and sensitivity when compared to T2-STIR and T2* sequences for the detection of IMH. However, further work may be needed before this can be used as a reliable surrogate end-point for clinical trials [125].

### CMR of microvascular obstruction and hemorrhage in myocardial infarction

Katherine Wu’s excellent review of MVO and hemorrhage in myocardial infarction summarized the underlying pathophysiology behind this common clinical problem as well as the CMR techniques used for its diagnosis, the clinical implications, and future directions needed for improving the understanding of the issues involved [126].

### Semi-automatic segmentation of myocardium at risk in T2-weighted cardiovascular magnetic resonance

Quantification of the area at risk (the region of viable but ischemic myocardium) after an acute coronary syndrome can be used to determine the amount of myocardial salvage following intervention. In a similar way to the assessment of late enhancement, there is debate as to the best measurement technique. As a result, studies have relied upon either manual delineation or threshold methods (using 2 standard deviations from remote regions, full width half maximum intensity, or Otsu’s method) but these techniques have inherent limitations. Sjögren et al. report an automatic segmentation algorithm for quantification of area at risk (AAR) which defines the continuous region most likely to represent AAR by estimating the intensities of normal myocardium and AAR with an expectation maximization algorithm, restricting the AAR region according to an a priori model of the maximal expected extent for the culprit artery [127]. In a study population of 47 STEMI patients, results were favourable with a good agreement between the automatic segmentation method and AAR assessed using manual delineation and all three threshold methods. Thus, this may represent a promising, objective method for standardized quantification of area at risk using T2-weighted sequences.

### Non-contrast T1-mapping detects acute myocardial edema with high diagnostic accuracy: a comparison to T2-weighted cardiovascular magnetic resonance

Although T2-weighted sequences have been widely used to assess myocardial oedema, quantitative T1-mapping is also sensitive to changes in free water content. The Oxford group in collaboration with Matthias Friedrich in Calgary compared 21 healthy controls and 21 patients with Takotsubo cardiomyopathy or acute regional myocardial edema without infarction. Wall motion, myocardial T1 values (ShMOLLI T1) and T2 signal intensity ratio relative to both skeletal muscle and remote myocardium (with both dark-blood T2-STIR and bright-blood ACUT2E) were compared on CMR scans performed at 1.5T within 7 days of the index event [128]. Receiver operator characteristics (ROC) analysis showed that T1-mapping had a significantly larger area-under-the-curve (AUC = 0.94) compared to T2-weighted methods, whether the reference ROI was skeletal muscle or remote myocardium (AUC = 0.58-0.89; p < 0.03). A T1 value of >990 ms most optimally differentiated segments affected by edema from normal segments, with a sensitivity and specificity of 92%. Non-contrast T1-mapping therefore may have a role as a complementary technique to T2-weighted imaging for assessing myocardial edema, such as quantifying area-at-risk and diagnosing myocarditis.

### Cardiovascular magnetic resonance of myocardial edema using a short inversion time inversion recovery (STIR) black-blood technique: diagnostic accuracy of visual and semi-quantitative assessment

In response to reportedly variable image quality associated with STIR sequences for the detection of myocardial oedema, the Leeds group studied image quality and diagnostic performance of STIR using a set of pulse sequence parameters dedicated to edema detection [129]. STIR images were evaluated acutely in STEMI patients for image quality as well as for presence and extent of myocardial hyper-intensity, with both visual and semi-quantitative (threshold-based) analysis. The extent of late enhancement was used as a reference standard for localization and extent of myocardial necrosis (acute) or scar (chronic). STIR image quality was rated as diagnostic in 99.5% of cases and reliably detected infarcted segments in the acute stage both on visual and semi-quantitative assessment. STIR also accurately differentiated acutely from chronically infarcted segments with a specificity of 99% by visual assessment and 97% by semi-quantitative assessment and with a sensitivity of 95% by both methods. The extent of hyper-intense areas on acute STIR images was 85% larger than those on LGE images, with a larger myocardial salvage index in reperfused than in non-reperfused infarcts (p = 0.035). The group concluded that with appropriate pulse sequence settings, T2-STIR is accurate in detecting acute myocardial infarction (MI) and distinguishing acute from chronic MI with both visual and semi-quantitative analysis. However, due to its unique technical characteristics, STIR should be regarded as an edema-weighted rather than a purely T2-weighted technique.

### Cardiovascular magnetic resonance by non contrast T1-mapping allows assessment of severity of injury in acute myocardial infarction

In a paper closely related to the group’s previous manuscript on the assessment of myocardial oedema, Dall’Armellina et al. reported the utility of pre-contrast T1-mapping for delineating the extent of ischemic injury and myocardial damage in the setting of acute myocardial infarction [130]. In 41 patients with acute myocardial infarction (78% STEMI), T2W, T1-mapping and LGE sequences were performed using 3T CMR both 12–48 hour after chest pain onset and at 6 months. Patients with STEMI underwent primary PCI prior to CMR. It was found that the variability of T1 measurements was significantly lower compared to T2W sequences and in terms of diagnostic performance, T1-mapping was superior to T2W imaging in NSTEMI. In STEMI patients, it was at least as good as T2W-CMR. The myocardial salvage index derived from acute T1-mapping and 6M LGE was no different to the one derived from T2W images (P = 0.88). Furthermore, the likelihood of improvement in segmental function at 6 months decreased progressively as acute T1 values increased (P < 0.0004). It therefore appears that T1-mapping might become an important complementary technique to LGE and T2W for identification of reversible myocardial injury and prediction of functional recovery in acute MI.

### Multicontrast delayed enhancement (MCODE) improves detection of subendocardial myocardial infarction by late gadolinium enhancement cardiovascular magnetic resonance: a clinical validation study

MultiContrast Delayed Enhancement (MCODE) imaging simultaneously provides a T2-weighted image with a phase sensitive inversion recovery (PSIR) LGE image and can be used to discriminate subendocardial infarction more clearly. With standard LGE techniques, this can be challenging especially if there is poor contrast between the blood pool and the infarct itself. Bandettini and colleagues reported a prospective comparison between MCODE and standard PSIR LGE imaging in 73 patients referred for vasodilator stress perfusion by targeted, repeat imaging of slice locations [131]. MCODE had similar specificity compared to LGE but demonstrated better sensitivity compared to LGE performed early and immediately before and after the MCODE (p = 0.008 and 0.02 respectively). While both PSIR LGE and MCODE were good at identifying MI, MCODE identified more subendocardial infarcts and a larger number of infarcted sectors than PSIR LGE alone. Thus the simultaneous acquisition of T1 and T2-weighted images improved the differentiation of blood pool from the enhancement associated with subendocardial MI and may prove very useful in clinical practice.

### In vivo chronic myocardial infarction characterization by spin locked cardiovascular magnetic resonance

Characterization of infarcted myocardium using CMR typically relies on late gadolinium enhancement (LGE) techniques as T1 and T2 weighted imaging is relatively insensitive to chronic infarction without the use of a paramagnetic contrast agent. Witschey et al. proposed that T1ρ-weighted rotating frame CMR techniques could be used to measure infarct size in the absence of a contrast agent due to the relatively large change in T1ρ relaxation time between scar and myocardium [132]. In a porcine infarct model, T1ρ-weighted imaging was performed with a B1 = 500 Hz spin lock pulse on a 3 T clinical MR scanner. T1ρ relaxation time maps were computed from multiple T1ρ-weighted images at varying spin lock durations. There was no difference in infarct size measured by high resolution late enhancement, T1ρ CMR or optical planimetry in the explanted hearts following sacrifice. T1ρ relaxation times were 91.7 ms in the infarct and 47.2 ms in remote myocardium, enabling good discrimination between infarcted and normal myocardium. The authors concluded that T1ρ CMR has the potential for visualizing MI without the need for exogenous contrast agents in a wide range of clinical cardiac applications.

### Cardiovascular magnetic resonance characterization of peri-infarct zone remodeling following myocardial infarction

At the edges of a region of infarcted myocardium, the peri-infarct zone (PIZ) contains a mixture of viable and non-viable myocytes, the extent of which is associated with susceptibility to arrhythmia (ventricular tachycardia) and adverse cardiac outcomes. In this study, the group attempted to characterize early temporal changes in scar morphology and regional function in the PIZ. Custom signal density threshold algorithms, based on remote myocardium, were applied to define infarct core and peri-infarct zone in minipigs with reperfused, anteroseptal infarcts [133]. The authors found that, after the initial edema following reperfused MI subsides, the peri-infarct zone remains dynamic and decreases in mass. The tensile forces in the PIZ also change as the infarct zone expands and thins out (with regional circumferential strain increasing between the infarct scar and the PIZ at 30 days post-MI). It is therefore hypothesised that remodelling characteristics of the PIZ might provide mechanistic insights into the development of life-threatening arrhythmias and sudden cardiac death post-MI.

### Infarct healing is a dynamic process following acute myocardial infarction

Following on from the previous paper, the dynamic nature of infarct healing in terms of the amount, the rate and duration was assessed in 66 patients enrolled following reperfusion for acute STEMI [134]. Mean infarct size decreased progressively from baseline (within 1 week of the acute event) to 4 months and 14 months, the largest infarcts having the greatest absolute decrease in mass. The percent reduction of infarct mass was independent of initial infarct size with a 32% decrease in infarct mass between acute necrosis and 4 months (p < 0.01) and an additional 12% decrease in infarct mass between 4 and 14 months (p < 0.01). This confirms that infarct healing is a continuous process after reperfusion for STEMI and that therefore, both decisions based on and interventions performed to reduce infarct size, must take into consideration the time frame of measurement.

### Remodeling after acute myocardial infarction: mapping ventricular dilatation using three dimensional CMR image registration

O’Regan et al. used 3D co-registration of CMR images to assess the long-term effects of ischemia-reperfusion injury on left ventricular structure in 46 patients following primary PCI for acute STEMI [135]. Patients were scanned within 7 days and again at one year. Localised LV wall changes were assessed using intensity-based similarities to track the structural changes in the heart between baseline and follow-up. As expected, local LV remodeling was greater within infarcted myocardium than in non-infarcted myocardium and was directly related to the transmural extent of infarction with greatest wall dilatation observed when infarct transmurality exceeded 50%. In addition, infarct remodeling was more severe when microvascular obstruction was present but only a modest dilatation was observed within non-ischemic myocardium. This technique therefore has potential for assessing dynamic regional changes in ventricular structure in relation to therapeutic interventions.

### Heme arginate improves reperfusion patterns after ischemia: a randomized, placebo-controlled trial in healthy male subjects

In this study, 3T blood oxygen level dependent (BOLD) functional MRI was used to assess whether heme arginate could prevent ischemia-reperfusion injury in the calf muscles of 12 healthy male subjects [136]. BOLD can measure changes in tissue oxygenation with a high spatial and temporal resolution. Heme arginate (1 mg/kg body weight) or placebo was infused 24 hours prior to a 20 minute period of leg ischemia induced by a thigh cuff. The peak reactive hyperemia signal of the calf muscles was significantly increased and occurred earlier after heme arginate than placebo. It is therefore possible that heme arginate may protect tissue against ischemia-reperfusion injury. The underlying mechanism is via induction of heme oxygenase-1. MR is able to provide this information using very simple, economical equipment both for calf muscle exercise within the magnet bore and for models of ischemia.

### Assessment of distribution and evolution of mechanical dyssynchrony in a porcine model of myocardial infarction by cardiovascular magnetic resonance

Cine, tagging and late gadolinium sequences were used in this study to characterize cardiac morphology, location and extent of MI, and regional mechanics in 6 pigs pre- and post-MI [137]. This was correlated with electro-anatomic mapping (EAM) performed within 24 hrs of CMR and prior to sacrifice. The authors found a significant decrease in global circumferential strain post MI with no significant change in peri-infarct and MI segments between early and late time-points (9 ± 2 vs. 22 ± 10 days). Time to peak strain (TTP) was significantly longer and its standard deviation greater in infarcted, compared to normal and peri-infarct segments, both early and late post-MI. EAM revealed late electrical activation and greatly diminished conduction velocity in the infarct when compared to peri-infarct and remote myocardium. From these data, the authors conclude that mechanical dyssynchrony occurs early after MI and is the result of delayed electrical and mechanical activation in the infarct. Mechanical dyssynchrony post-MI is associated with adverse LV remodelling and increased mortality. Interventions to minimise infarct size may therefore have beneficial effects on survival.

### A CMR study of the effects of tissue edema and necrosis on left ventricular dyssynchrony in acute myocardial infarction: implications for cardiac resynchronization therapy

CMR is known to have utility in guiding resynchronization therapy [138,139]. In this study, a novel 3D tagging sequence was used to produce the ‘CURE’ index (circumferential uniformity ratio estimate; a value of 1 = complete synchrony) in 22 patients following successful PCI. Edema (using T2-weighted images) and necrosis (using late enhancement) were quantified to determine the region of salvaged myocardium (area-at-risk minus necrosis) [140]. The authors found that in the acute phase, the extent of edema correlated with dyssynchrony, while extent of necrosis showed only a borderline correlation. PCI resulted in salvaged myocardium of 27 ± 14%. LV dyssynchrony (using the CURE index) improved at 4 months from 0.91 ± 0.05 to 0.94 ± 0.03 (p < 0.004). At 4 months, edema was absent and LV scar percentage had reduced slightly. Although LV dyssynchrony was closely related to the extent of edema in the acute phase of infarction, necrosis was found to be a poor predictor of acute dyssynchrony. However, reduction in intraventricular dyssynchrony during infarct healing was predicted by both the extent of edema and necrosis in the acute phase. This study is a further demonstration of the utility of CMR for assessing surrogate markers of adverse outcome in acute infarction.

### Thrombus aspiration during primary percutaneous coronary intervention is associated with reduced myocardial edema, hemorrhage, microvascular obstruction and left ventricular remodeling

It is established that MVO and hemorrhage portend an adverse clinical outcome and it therefore follows that strategies aimed at minimizing these might result in an improved prognosis. In this retrospective study, Zia showed that coronary thrombus aspiration (TA) during primary percutaneous coronary intervention (PCI) was associated with reduced myocardial edema, myocardial hemorrhage, left ventricular remodeling and incidence of MVO after STEMI (assessed by T2, T2*, steady-state free precession cine and contrast-enhanced T1-weighted inversion recovery gradient-echo sequences respectively) [141]. Sixty patients, enrolled after primary PCI, underwent CMR scans at 48 hours and 6 months. They were retrospectively stratified into 2 groups: those that received TA versus those that did not. At 48 hours, infarct segment T2 was lower (p = 0.022), indicative of less edema, infarct segment T2* was higher (p = 0.007), suggesting a lesser degree of myocardial hemorrhage and the incidence of MVO was lower (p = 0.013) in the TA group. In this same group at six months, the LV end-diastolic volume index and LV end systolic volume index were lower (p = 0.013 and p = 0.008 respectively) and the infarct segment systolic wall thickening was higher (p = 0.003). Although this intuitively appears a very positive result, a degree of caution is needed in interpreting the results at face value. In a retrospective subgroup analysis of the HORIZONS-AMI study, the use of thrombus aspiration in STEMI patients undergoing primary PCI was associated with improved ST-segment resolution at discharge, but not with any difference in final coronary artery flow, myocardial perfusion (assessed by myocardial perfusion grade at cardiac catheterisation) or major adverse cardiac events (MACE) [142].

## Gadolinium use in CMR

The power of CMR is in no small part due to the additive information that can be gained by the administration of gadolinium based contrast agents (GBCAs), and these are being applied in new situations especially the atria [143]. There is now general awareness of the rare but potentially serious complication of nephrogenic systemic fibrosis (NSF) observed following administration of some earlier GBCAs, notably in the presence of significant renal impairment. This had led to guidelines regarding the use of these agents.

### Gadolinium in pediatric cardiovascular magnetic resonance: what we know and how we practice

No GBCA is currently approved for use in children under the age of 2 years. This manuscript reports the results of the 70 replies to a worldwide survey of GBCA use by cardiac imagers with paediatric experience, with an emphasis on studying the use of GBCAs in neonates [144]. The predominant agent used was gadopentetate dimeglumine (by 49% of respondents). Choice was principally determined by availability with some consideration to licensed indications above side effect profile or image quality. Use of GBCAs in neonates by respondents was widespread (93%) with 79% using agents in neonates of <1 week of age. No respondents administered GBCAs if the estimated glomerular filtration rate was below 30 ml/min/1.73m^2^. Nausea or vomiting were reported as common side effects by 60% of respondents. The manuscript supports awareness by responding cardiac imagers of the immature renal function and physiological differences in paediatric patients that places them at risk of NSF. It also highlights the paucity of data regarding use of such agents in the neonatal and paediatric populations, and the resulting hindrance to licensing and guideline formulation.

### Gadolinium-enhanced cardiovascular magnetic resonance: administered dose in relationship to united states food and drug administration (FDA) guidelines

Growing awareness of the relationship between gadolinium exposure and the development of NSF led to revised label warnings for GBCAs in 2008. The results of a meta-analysis of gadolinium dosing regimes in 233 peer-reviewed studies published between 2004 and 2010 where gadolinium was employed for CMR in a total of 19,934 patients are reported in this manuscript [145]. In 199 of the studies, the indication for gadolinium administration was in the assessment of late myocardial enhancement. Almost half of the studies (114) used gadopentetate dimeglumine. The analysis shows that in the published literature gadolinium is typically administered at higher than label-recommended doses (with median doses for each year 0.15 mmol/kg or more) and that no significant dose reduction (P > 0.05) was seen following FDA black box warnings. The manuscript highlights the need for clinical trials to determine the most appropriate doses of gadolinium for CMR studies.

### Minimizing risk of nephrogenic systemic fibrosis in cardiovascular magnetic resonance

Awareness of NSF and the pre-requisite substrate of severe renal impairment for developing this condition, together with more stable gadolinium chelates has created a somewhat self-limiting problem. Nevertheless, this review elegantly provides an overview of what remains a relevant issue, notably with respect to maintaining the safe use of GBCAs [146]. The ongoing prevention of cases remains important given there is no cure for NSF. The review describes the current understanding of NSF pathochemistry, pathogenesis and treatment options, with an emphasis on translating the various guidelines into clinical practice so as to negate the risk of NSF.

## Myocardial mechanics

Measuring cardiac function is a fundamental for CMR. Myocardial tagging has recently been reviewed [147], and newer techniques have been reported in humans [148,149] and animals. [150,151] feature tracking has become an area of active interest with development of simple to use software that can be used post-hoc on simple cines, however the lack of validation remains an issue. The papers pursue novel aspects of cardiac function.

### Quantification of biventricular myocardial function using cardiac magnetic resonance feature tracking, endocardial border delineation and echocardiographic speckle tracking in patients with repaired tetralogy of Fallot and healthy controls

Feature tracking (FT) post processing of routinely acquired SSFP cines is an appealing approach to quantification of myocardial strain, and has been used during dobutamine stress [152]. This study applied it to the RV as well as the LV, which is of potential interest as CMR allows more complete RV wall coverage than echocardiography [153]. While FT software provides readouts of regional strain, these have yet to be validated. In this study, even the inter-observer reproducibility of regional FT analyses of the same cine acquisitions appeared suboptimal. Inter-study reproducibility was not investigated. Global measures of strain by FT showed more encouraging levels of inter-observer reproducibility and of agreement with comparable measures by echocardiographic speckle tracking in the same patients. Interestingly, however, the authors’ own endocardial boundary tracking software, which they programmed using Matlab, gave comparable levels of reproducibility and agreement, implying that measures of global circumferential strain by FT may represent little more than measures of diastolic-systolic change of cavity boundary length.

### Inter-study reproducibility of cardiovascular magnetic resonance myocardial feature tracking

This paper reported the inter-study variation of global and regional strain as measured by feature tracking analyses of CMR SSFP cines in 16 healthy volunteers [154]. Although this was not attempted in patients with ventricular dysfunction, and was not compared here with any reference method such as tagging, inter-study reproducibility is clearly more clinically relevant than inter-observer reproducibility, which says little about the reproducibility in practice of an image post-processing method. The inter-study reproducibility of different strain analyses varied. The coefficient of variation (CV) of global assessment by FT of circumferential strain in short axis slices was 20.3%, compared with 38% for segmental analysis of the same. The least reproducible segmental measure was that of right ventricular longitudinal strain (CV 60%) whilst the least reproducible global measure was that of radially directed strain measured from 4-chamber long axis cines (CV 33.3%). CMR FT may have potential for quantitative wall motion analysis, for example in clinical trials but, given the coefficients of variation found in volunteers, users should clearly interpret comparable FT measurements individual patients with caution.

### Undersampled cine 3D tagging for rapid assessment of cardiac motion

This study reported k-t undersampled cine 3D tagging in conjunction with k-t principal component analysis (PCA) reconstruction for single breath-hold acquisitions of multidirectional strain of the whole heart [155]. The performance of such undersampled methods was investigated using computer simulations and in-vivo measurements in 8 healthy subjects and 5 patients with myocardial infarction. In patients, peak circumferential shortening was reduced in regions with late gadolinium enhancement. The undersampled approach allowed reduction in acquisition time of whole-heart tagging which facilitated quantification of shortening, rotation and torsion of the left ventricle without adding significant errors compared to previous 3D tagging approaches.

### Evaluation of left ventricular torsion by cardiovascular magnetic resonance

This paper reviews current clinical applications LV torsion measurements by CMR, showing how torsion can give insights into LV mechanics and the influence of LV geometry and myocyte fiber architecture on cardiac function [156]. It offers recommendations for CMR measurement protocols, attempts to stimulate standardization of torsion calculation, and suggests areas for future research.

### Subendocardial contractile impairment in chronic ischemic myocardium: assessment by strain analysis of 3T tagged CMR

This study reported measurements of myocardial strain in the subendocardial and epicardial layers of the LV by resting tagged CMR at 3T in 12 patients with severe chronic coronary artery disease who had been scheduled for coronary artery bypass grafting [157]. Myocardial segments were divided into stenotic and non-stenotic by invasive coronary angiography, and ischemic and non-ischemic segments by stress myocardial perfusion scintigraphy. Analyses showed lower circumferential stain in stenotic than non-stenotic segments, without significant differences between the two groups for strain of the epicardial layer. They also showed impairment of circumferential strain in the subendocardial layer of chronically ischemic myocardium at rest.

### Mapping right ventricular myocardial mechanics using 3D cine DENSE cardiovascular magnetic resonance

This study reports measurements of RV mechanics by whole heart three dimensional cine displacement encoding with stimulated echoes (DENSE) CMR in 5 healthy volunteers [158]. The inflow region showed lower peak strains than the apical and outflow regions, and the time to peak strains suggested RV mechanical activation in the order: inflow, outflow, mid, then apex. The techniques used may have clinical utility for assessing altered RV mechanics in disease.

### Three-dimensional regional strain analysis in porcine myocardial infarction: a 3T magnetic resonance tagging study

This study set out to investigate the incremental value of 3D relative to 1D or 2D harmonic phase analyses of strain by CMR in the assessment of viability in a porcine model of infarction [159]. The 3D strain information distinguished infarcts and their neighboring regions from healthy myocardium. The 3D interrogation of contractility was found to give incremental diagnostic accuracy in delineating the dysfunctional and nonviable myocardium relative to 1D or 2D methods. The infarct neighboring regions were the major beneficiaries of the more comprehensive, 3D assessment of regional strain.

## Left ventricular volumes and motion

Given increasing availability and uptake of CMR, it is ever more important to determine the appropriate application of CMR derived values to clinical use. Although there is correlation between ejection fraction measure by CMR and echocardiography, the values are not interchangeable. Consequently, where an evidence base has been established in one modality, the threshold value cannot necessarily accurately and reliably be transferred across modalities. Additional CMR derived parameters continue to be generated, increasingly from the acquisition of 3D data sets. An emphasis on expanding the collection of robust prospectively acquired multi-centre multi-vendor scientific data is required to generate appropriate clinical and prognostic thresholds for CMR derived parameters. Although the fundamental techniques for measuring ventricular function and mass using the contiguous parallel short axis slices techniques are well established, there are continuing reports of variants of the basic technique [160], and extension of its application to all 4 cardiac chambers [161,162]. The excellent reproducibility of left ventricular mass measurements makes CMR valuable in studies of drug efficacy [163].

### Potential clinical impact of cardiovascular magnetic resonance assessment of ejection fraction on eligibility for cardioverter defibrillator implantation

This retrospective single centre study of 52 patients highlights the clinically important discrepancy between left ventricular ejection fraction (LVEF) as measured by CMR compared to echocardiography and investigates the potential impact of applying the echo derived selection criteria for ICD implantation to CMR derived data [164]. The use of CMR derived LVEF against the echocardiographic derived threshold of 35% would have resulted in 21% of patients being reclassified with regards to ICD eligibility. Of further note, CMR identified the presence of LV thrombus in 6 of the patients under consideration for ICD implantation, whereas echocardiography only identified 1 of these. The argument for CMR to be the imaging modality of choice in assessing device suitability is strengthened by the superior reproducibility of CMR compared to echocardiography, let alone its potential for viability and scar imaging. Large scale multi-centre prospective studies are required to better define the clinical evidence base for CMR parameters further.

### Late gadolinium enhancement by cardiovascular magnetic resonance is complementary to left ventricle ejection fraction in predicting prognosis of patients with stable coronary artery disease

In this study, 376 patients with stable coronary artery disease were followed up prospectively for 38 ± 21 months to assess the incremental prognostic value of late gadolinium enhancement (LGE) as an adverse prognostic factor above conventional risk factors including LVEF [165]. LGE and LVEF were both robust independent non-invasive markers of prognosis with respect to the primary end point of all cause mortality and hospitalisation for a new diagnosis of heart failure (HR: 13.61 for LGE ≥ 45% of LV mass; and 12.34 for LVEF ≤ 30%; p < 0.0001). Furthermore LGE enhanced risk stratification when incorporated into a multivariate model.

### A quantitative comparison of regional myocardial motion in mice, rabbits and humans using in-vivo phase contrast CMR

This manuscript addresses the important issue of how closely small animal myocardial motion resembles and replicates human cardiac physiology and pathology [166]. Using phase contrast CMR to assess regional 3D left ventricular motion with high temporal resolution in 18 mice, 8 rabbits and 9 humans, subtle but significantly different regional myocardial motion patterns were observed between and within species. Given the prominent role of genetically modified animal models in researching human cardiovascular disease, these differences must be considered.

### Volumetric motion quantification by 3D tissue phase mapped CMR

This manuscript describes extensive quantification of myocardial motion of the entire left ventricle from 3D tissue phase mapped (TPM) CMR in 12 healthy volunteers and 2 patients [167]. Having shown in a previous publication (JCMR 2011) that SENSE can be used to accelerate 2D TPM, here the authors use SENSE to facilitate 3D acquisitions with isotropic pixel size. They propose that this facilitates more detailed analysis of LV motion which may enhance patient selection for cardiac resynchronisation therapy. It was possible to determine velocity, torsion, rotation and strain derived parameters from the 3D-TPM data, and also identify abnormal wall motion in the patients compared to the healthy subjects using these. Additional data will be required to more fully elucidate the clinical prognostic value of these parameters.

## Varia

There are always papers which defy simple categorization. This section pulls together such varia which includes society reports [168], and novel [169], or unusual techniques [170-172].

### Review of journal of cardiovascular magnetic resonance 2011

The review of JCMR papers in 2011 [173], and the President's page [174], were highly accessed which the editors consider reflects the interest of the readership in having a review article collating the year's papers bringing together the articles into thematic headings.

### Assessment of MRI issues at 3-tesla for metallic surgical implants: findings applied to 61 additional skin closure staples and vessel ligation clips

Metallic skin closure staples and vessel ligation clips were assessed at 3 Tesla for magnetic field interactions, heating, and artifacts in this paper [175]. MRI-related heating was assessed by placing each implant in a gelled-saline-filled phantom with MRI performed using a transmit/receive RF body coil at an MR system reported, whole body averaged SAR of 2.9-W/kg for 15-min. Artifacts were characterized using T1-weighted, SE and GRE pulse sequences. Each surgical implant showed minor magnetic field interactions (20- and 27-degrees, which is acceptable from a safety consideration). Heating was not substantial (highest temperature change, ≤ 1.6°C). Artifacts may create issues if the area of interest is in the same area or close to the respective surgical implant.

Cost evaluation of cardiovascular magnetic resonance versus coronary angiography for the diagnostic work-up of coronary artery disease: application of the european cardiovascular magnetic resonance registry data to the german, united kingdom, swiss, and united states health care systems.

CMR has favorable characteristics for diagnostic evaluation and risk stratification of patients with known or suspected CAD. CMR utilization in CAD detection is growing fast. However, data on its cost-effectiveness are scarce. The goal of this study was to compare the costs of two strategies for detection of significant coronary artery stenoses in patients with suspected coronary artery disease (CAD): 1) Performing CMR first to assess myocardial ischemia and/or infarct scar before referring positive patients (defined as presence of ischemia and/or infarct scar to coronary angiography (CXA) versus 2) a hypothetical CXA performed in all patients as a single test to detect CAD [176]. A subgroup of the European CMR pilot registry was used including 2,717 consecutive patients who underwent stress-CMR. From these patients, 21% were positive for CAD (ischemia and/or infarct scar), 73% negative, and 6% uncertain and underwent additional testing. The diagnostic costs were evaluated using invoicing costs of each test performed. Costs analysis was performed from a health care payer perspective in German, United Kingdom, Swiss, and United States health care settings. In the public sectors of the German, United Kingdom, and Swiss health care systems, cost savings from the CMR-driven strategy were 50%, 25% and 23%, respectively, versus outpatient CXA. If CXA was carried out as an inpatient procedure, cost savings were 46%, 50% and 48%, respectively. In the United States context, cost savings were 51% when compared with inpatient CXA, but higher for CMR by 8% versus outpatient CXA. The authors conclude that the use of CMR should be encouraged as a management option for patients with suspected CAD.

### Monocenter feasibility study of the MRI compatibility of the evia pacemaker in combination with safio s pacemaker lead

The number of MR conditional pacemakers is increasing, which has been reviewed recently [177], and reports are now emerging of performance of imaging of the heart by MR in their presence [178]. The purpose of this study was to evaluate the feasibility of the magnetic resonance (MR) conditional pacemaker (PM) system (Evia SR-T and DR-T with Safio S leads) under MR conditions [179]. Patients with standard PM indications and Evia PM were eligible for enrollment in this single center prospective non-randomized pilot study. Patients underwent MR of the brain and lower lumbar spine at 1.5 Tesla. Atrial (RA) und ventricular (RV) lead parameters (sensing, pacing threshold [PTH], pacing impedance) were assessed immediately before (baseline follow-up [FU]) and immediately after MRI (1st FU), after 1 month (2nd FU) and 3 months (3rd FU). The effect of MR on serious adverse device effect (SADE) free-rate, on atrial and ventricular sensing (AS/VS; mV) and atrial (RA) and ventricular (RV) pacing thresholds (PTH; V/0.4 ms) were investigated between baseline and 2nd FU. Data of 30 patients (female 12 [40%], age 73 ± 12 years, dual chamber PM 15 [50%]) were included in this analysis. No MR related SADE occurred. Lead measurements were not statistically different between the baseline FU and the 2nd FU (AS/VS at baseline 3.2 ± 2.1/15.0 ± 6.0, at 2nd FU 3.2 ± 2.1/14.9 ± 6.5; p = ns. RA-PTH/RV-PTH at baseline 0.68 ± 0.18/0.78 ± 0.22, at 2nd FU 0.71 ± 0.24/0.78 ± 0.22; p = ns). The presence of the permanent pacemakers led to MR imaging artifacts on diffusion weighted sequences of the brain, but did not affect other sequences (e.g. FLAIR and T2 weighted spin-echo images). The authors conclude that the use of the MR conditional Evia PM in a MR environment under predefined conditions is feasible.

### Cardiovascular magnetic resonance physics for clinicians: part II

This is the second of 2 reviews [180], covering the essential aspects of CMR physics in a way that is understandable and relevant to clinicians using CMR in their daily practice. Starting with the basic pulse sequences and contrast mechanisms described in part I, it briefly discusses further approaches to accelerate image acquisition. It then continues by showing in detail how the contrast behaviour of black blood fast spin echo and bright blood cine gradient echo techniques can be modified by adding rf preparation pulses to derive a number of more specialised pulse sequences. The simplest examples described include T2-weighted oedema imaging, fat suppression and myocardial tagging cine pulse sequences. Two further important derivatives of the gradient echo pulse sequence, obtained by adding preparation pulses, are used in combination with the administration of a gadolinium-based contrast agent for myocardial perfusion imaging and the assessment of myocardial tissue viability using a late gadolinium enhancement (LGE) technique. These two imaging techniques are discussed in more detail, outlining the basic principles of each pulse sequence, the practical steps required to achieve the best results in a clinical setting and, in the case of perfusion, explaining some of the factors that influence current approaches to perfusion image analysis. The key principles of contrast-enhanced magnetic resonance angiography (MRA) are also explained in detail, especially focusing on timing of the acquisition following contrast agent bolus administration, and current approaches to achieving time resolved MRA. Alternative MRA techniques that do not require the use of an endogenous contrast agent are summarised, and the specialised pulse sequence used to image the coronary arteries, using respiratory navigator gating, is described in detail. The article concludes by explaining the principle behind phase contrast imaging techniques which create images that represent the phase of the MR signal rather than the magnitude. It is shown how this principle can be used to generate velocity maps by designing gradient waveforms that give rise to a relative phase change that is proportional to velocity and the choice of velocity encoding range and key pitfalls in the use of this technique are discussed.

## Competing interests

The authors declare that they have no competing interests.

## Authors’ contributions

All authors contributed to the writing of this review article. All authors read and approved the final manuscript.
